# Effects of *Artemisia annua* and *Foeniculum vulgare* on chickens highly infected with *Eimeria tenella* (Phylum *Apicomplexa*)

**DOI:** 10.1186/1751-0147-56-22

**Published:** 2014-04-15

**Authors:** Liviu Drăgan, Adriana Györke, Jorge FS Ferreira, Ioan A Pop, Ioan Dunca, Maria Drăgan, Viorica Mircean, Iosif Dan, Vasile Cozma

**Affiliations:** 1 Veterinary and Food Safety Unit Mureş, Animal Health Service, 10 Podeni Street, 540253 Târgu Mureş, Romania; 2 Faculty of Veterinary Medicine, Parasitology and Parasitic Diseases Department, University of Agricultural Science and Veterinary Medicine Cluj Napoca, 3-5 Calea Mănăştur, 400372 Cluj-Napoca, Romania; 3 US Salinity Laboratory (USDA-ARS), 450 W. Big Springs Road, Riverside, CA 92507, USA; 4 Agency of Reproduction in Animal Breeding Mureş, 76 Budiului Street, 540390 Târgu Mureş, Romania; 5 S.C. Transapicola S.R.L., 547365 Livezeni 101, Mureş, Romania

**Keywords:** *Eimeria tenella*, *Artemisia annua*, *Foeniculum vulgare*, Anticoccidial effect

## Abstract

**Background:**

Intensive poultry production systems depend on chemoprophylaxis with anticoccidial drugs to combat infection. A floor-pen study was conducted to evaluate the anticoccidial effect of *Artemisia annua* and *Foeniculum vulgare* on *Eimeria tenella* infection. Five experimental groups were established: negative control (untreated, unchallenged); positive control (untreated, challenged); a group medicated with 125 ppm lasalocid and challenged; a group medicated with *A. annua* leaf powder at 1.5% in feed and challenged; and a group treated with the mixed oils of *A. annua* and *Foeniculum vulgare* in equal parts, 7.5% in water and challenged. The effects of *A. annua* and oil extract of *A. annua + F. vulgare* on *E. tenella* infection were assessed by clinical signs, mortality, fecal oocyst output, faeces, lesion score, weight gain, and feed conversion.

**Results:**

Clinical signs were noticed only in three chickens from the lasalocid group, six from the *A. annua* group, and nine from the *A. annua + F. vulgare* group, but were present in 19 infected chickens from the positive control group. Bloody diarrhea was registered in only two chickens from *A. annua* group, but in 17 chickens from the positive control group. Mortality also occurred in the positive control group (7/20). Chickens treated with *A. annua* had a significant reduction in faecal oocysts (95.6%; *P* = 0.027) and in lesion score (56.3%; *P* = 0.005) when compared to the positive control. At the end of experiment, chickens treated with *A. annua* leaf powder had the highest body weight gain (68.2 g/day), after the negative control group, and the best feed conversion (1.85) among all experimental groups.

**Conclusions:**

Our results suggest that *A. annua* leaf powder (Aa-p), at 1.5% of the daily diet post-infection, can be a valuable alternative for synthetic coccidiostats, such as lasalocid.

## Background

Avian coccidiosis is one of the most economically important diseases of the poultry industry, caused by apicomplexan parasites belonging to the genus *Eimeria*. There are seven species in this genus that affect chickens, with *E. tenella* being one of the most pathogenic [1]. Infection with *E. tenella* is followed by caecal lesions (petechiae, thickening, ecchymoses, accumulation of blood and caseous necrotic material in the caecum), accompanied with bloody diarrhea [2].

Intensive poultry production systems depend on chemoprophylaxis with anticoccidial drugs to combat infection. Anticoccidial drugs have been used for over 60 years, and their extensive use has led to the development of drug-resistant *Eimeria* spp. strains [3-5]. Drug-resistant strains are responsible for subclinical coccidiosis and economic losses due to poor weight gain and high food consumption. It was estimated that the economic losses in India, from 2003 to 2004, were 68.08% related to reduced body weight gain and 22.7% related to increased feed conversion ratio (FCR) [6].

Based on the health and economic damages mentioned above, there is increasing interest in the development of alternative strategies for disease control and prevention in poultry [7], including new molecules and vaccines. As part of this effort, studies on the inhibitory effects of natural products on *Eimeria* and/or on the protective immunity of the host were recently published [8,9].

*Artemisia annua*, L. (Asteraceae) is a plant whose dried leaves have been used in traditional Chinese medicine for over 2 millennia [10]. Currently, extensive studies have demonstrated that artemisinin, the main bioactive sesquiterpene lactone from *A. annua,* exhibits high efficacy against several stages of *Plasmodium*[11]. Other studies have indicated that artemisinin and related drugs (artemisinins) are effective against other protozoan parasites. Examples include the combined use of arteether and buparvaquone against *Babesia equi* in donkeys [12], artemisinin effects against cutaneous leishmaniosis [13], partially linked to cell-cycle arrest and apoptosis [14], and the *in vitro* effect of artemisinins on trypanosomes, at micromolar concentrations, linked to the inhibition of calcium-dependent ATPase on the parasite membrane [15].

Regarding the anticoccidial effects of *A. annua* in chickens, past studies indicated that both artemisinin and *A. annua* can be effective against *Eimeria* spp. [16-19]. Regarding anthelmintic effects, artemisinin at 200 mg/kg and ethanolic extracts of *A. annua* at 600–1000 mg/kg, both provided for five days, were safe but unable to control the nematode *Haemonchus contortus* in a gerbil model [20], while the same ethanolic extracts were effective in killing the trematodes *Schistosoma japonicum* and *Fasciola hepatica in vitro* at 2.0 mg/mL [21]. These results indicate that the same extract had different results depending on the in vitro (trematodes) or in vivo model (*Haemonchus*-infected gerbils) used. Results can also vary with different strains of the same parasite.

*Foeniculum vulgare* (fennel), the other herb tested in our study, is a medicinal plant belonging to the family *Apiaceae* (*Umbelliferae*), which has been used in the Mediterranean region as an aromatic herb. The major components of the essential oil are phenylpropanoid derivatives and monoterpenoids [22-24]. Fennel is known to have hepatoprotective effects [25], antispasmodic effects [26], diuretic, anti-inflammatory, analgesic, antioxidant, antimicrobial, antifungal and anticancer activities [24,27]. However, to our knowledge, fennel essential oil has never been studied for antiparasitic activity.

In this study, we investigated the anticoccidial effects of *A. annua* (leaf powder) and of mixed oils of *F. vulgare* and *A. annua* when either leaf powder or essential oils were added to diets fed to chicks challenged with *E. tenella* (1×10^4^ oocysts) in a floor-pen trial.

## Methods

### Animals and management

One hundred, 1-day-old broiler chicks (breed Ross 308) were obtained from S.C. Oprea Avicom S.R.L. (Venchi-Sigişoara, Romania) hatchery. They were reared as a single group from one-day old to 8-day-old and housed in a coccidia-free environment at S.C. Transapicola S.R.L. (Târgu Mureş, Romania). At 8 days of age, chicks were vaccinated for Newcastle and, at 12 days of age, for Marek disease. Broilers were identified before assigning them to their respective experimental groups. Diet formulas contained no anticoccidial feed additives. Chicks were offered feed and water ad libitum, and were reared under continuous light.

### Parasites

*Eimeria tenella* (Houghton strain) was obtained from Veterinary Laboratory Agency (Parasitology Unit) New Haw, UK. Oocysts were propagated, isolated and sporulated using standard procedures [28] at the Parasitology and Parasitic Diseases Department of Veterinary Faculty from Cluj-Napoca, Romania. Each chick received challenge infections by gavage as a suspension of 1×10^4^ sporulated oocysts in a volume of 1 mL.

### Artemisia and foeniculum products

*Artemisia annua* and *F. vulgare* were used as (i) *A. annua* leaf powder as prophylactic treatment, and as (ii) mixed essential oils of *A. annua* and *F. vulgare* in equal parts as treatment*.* The essential oil of *A. annua* was obtained from vegetative and flowering organs and the essential oil of *F. vulgare* was obtained from achenes.

### Artemisia annua powder

*Artemisia annua* plant material used, named GERM07, was the cultivar A-3 from Anamed (Winnenden, Germany). Seeds were germinated in mid-March in a greenhouse, transplanted to the field in early June, and harvested in early October of 2007, when plants were in the late vegetative stage (Anamed A-3 hybrid is a late-flowering clone) in Livezeni, Târgu-Mureş (46.55° N, 24.63° E). Plants were dried for three weeks under shade, at ambient temperature (20°C). Then, leaves were manually separated from the stems, dried, and ground finely to obtain *A. annua* leaf powder. Broiler feed was prepared by adding 15 kg of *A. annua* GERM07 powder per ton of feed (1.5%), and fed daily to infected animals until the end of the experiment (35 days).

### Mixed essential oils of artemisia annua and foeniculum vulgare

*Artemisia annua* essential oil was obtained from a Romanian variety of *A. annua* (ROMN 08)*,* cultivated at the same site and under the same conditions as GERM07, but harvested when plants were in early to late blooming stage. The essential oil was obtained from vegetative and flowering parts (excluding main stems and branches) through steam distillation.

*F. vulgare* essential oil obtained from seeds of cultivated plants was purchased from Fares® 1929 (Romania). The chemical composition, according to manufacturer of *F. vulgare* essential oil was: 68% *trans*-Anethole, 18% fenchone, 2.5% methyl chavicol, 3% limonene, 5% α-pinene and traces of camphene, sabinene, β-pinene, myrcene, camphor, carvone, anisaldehyde and others.

An essential oil mixture containing 25% essential oil of *A. annua,* 25% essential oil of *F. vulgare*, and 50% Tween 20 were administered 7.5% in water.

### Lasalocid

Lasalocid (Avatec®150 G; Alpharma, Belgium) was administered, with the diet, two days before experimental infection at a concentration of 125 mg per kg of feed.

### HPLC-UV analysis of artemisia annua

Artemisinin, deoxyartemisinin, dihydroartemisinic acid, and artemisinic acid were extracted from 500 mg of leaves of the GERM 07 *A. annua* strain. Leaves were mixed with diatomaceous earth using a mortar and pestle, with enough diatomaceous earth to fill 10-mL stainless steel cells, and extracted with hexane as a solvent using an accelerated solvent extraction system (ASE 350, Dionex USA). Extraction temperature was 100°C, at 1,500 psi, for a static time of five minutes. The petroleum ether fraction was transferred to a fume hood, evaporated overnight and, next day, reconstituted in 20 mL of acetonitrile (two washes of 10 mL each). The acetonitrile extract was filtered through a 0.45 μm nylon filter attached to a 10-mL Luer lock syringe and transferred to a 20-mL scintillation vial. Samples of 10 μL were injected by the HPLC auto-sampler into the system (Agilent 1100 series). Artemisinin, dihydroartemisinic acid, and artemisinic acid were quantified by high-performance liquid chromatography with photodiode array detection (HPLC-UV), according to a published protocol [29].

### Experimental design

At eight days of age, chicks were randomly divided by their identification tag number into 5 groups of 20 birds each as follows: group *Neg* = uninfected and untreated control group (negative control); group *Pos* = infected and untreated control group (positive control); group *Las* = infected and treated with lasalocid; group *Aa-p* = infected and treated with *A. annua* GERM07 as ground leaves in feed; group *Aa + Fv-o* = infected and treated with an mixture containing the essential oils of *A. annua* ROMN08 and *F. vulgare*, mixed with Tween 20 (1:1:2), added to water at a 7.5% ratio (v/v). The experimental groups were placed in a house from S.C. Transapicola S.R.L. (Târgu Mureş), in coccidia-free pens measuring 130 × 130 × 250 cm. At 10-days-old chicks were infected with 1×10^4^*E. tenella* oocysts. Treatments with *A. annua* and *F. vulgare* started with infection day and continued untill the end of the study (35 days). Treatment with lasalocid started 2 days before infection and lasted for the entire duration of the experiment.

The experimental design was approved by the Ethics Committee of the Cluj Napoca University.

### Observations and analytical procedures

Efficacy of *A. annua* and *F. vulgare* against *E. tenella* infection was evaluated by (i) clinical signs, (ii) mortality rate, (iii) oocysts shed per gram of faeces (OPG) (McMaster counting technique), (iv) faeces and (v) lesion scores, (vi) body weight gain (BWG) and (vii) feed conversion ratio (FCR). For oocysts counting, pooled fecal samples were collected daily from 7 to 17 days post-challenge, and then from days 20, 25, 30, and 35 post-challenge from each group. Two days before (days 5, and 6 post-challenge), we analyzed feces samples by flotation technique to establish the starting day for oocysts counting. At each sampling time, 40 fresh droppings were collected per group and mixed, resulting in approxiamtely 200 g faeces per group. Sodium chloride (sp.gr. 1.20) solution was used as flotation solution, and three counts were made per day and per group.

Faecal score was evaluated daily from the 4^th^ to the 9^th^ day post-infection and scored on a scale of 0–4, according to the consistency of the droppings and the presence of mucus/ blood (0 = normal droppings; 1 = normal to pasty; 2 = liquid; 3 = liquid with blood; 4 = bloody droppings, absence of normal fecal consistency).

Lesion score in the gut was evaluated at 7 days (10 chicks) post-challenge using a score of 0–4 [30].

### Statistical analysis

As our quantitative data for OPG and BWG did not follow a normal distribution in all groups; we proceeded with the statistical analysis of log-transformed data. Statistical analysis was made with Mann–Whitney U, and Kruskal-Wallis tests for group comparison using MedCalc software. A *p* value ≤0.05 was considered statistically significant.

## Results

### HPLC-UV analysis of A. annua leaf powder

**Figure 1:**
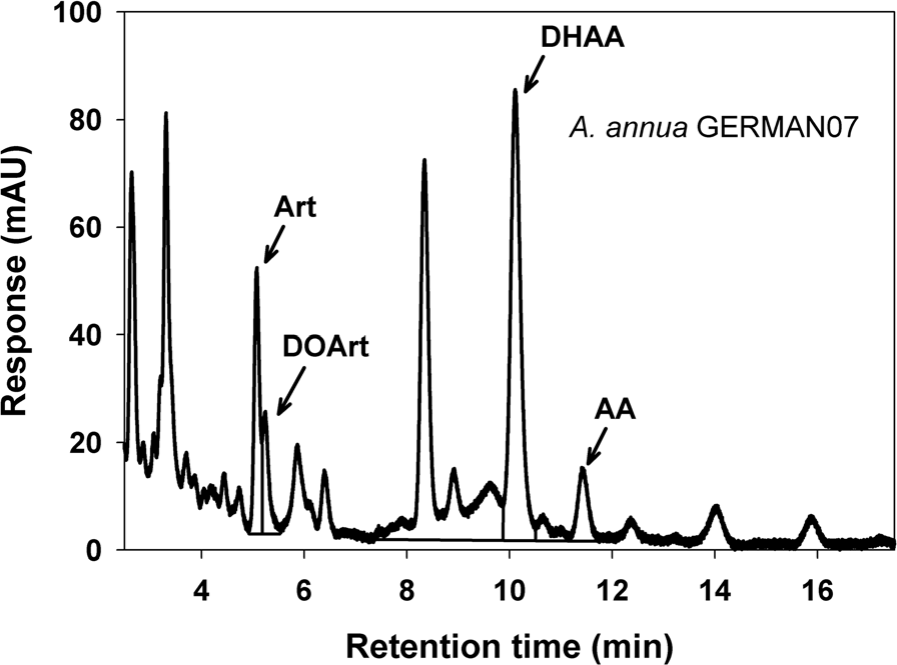
**HPLC-UV (192 nm) chromatogram of a 10-μL injection of *A. annua *(GERMAN07) leaf powder extract.** Quantification (in g/100 g dry weight) determined that artemisinin (Art), dihydroartemisinic acid (DHAA), and artemisinic acid (AA) were present at 0.75, 0.18, and 0.03%, respectively. Deoxyartemisinin (DOArt) was also present but has no reported biological activity.

**Figure 2:**
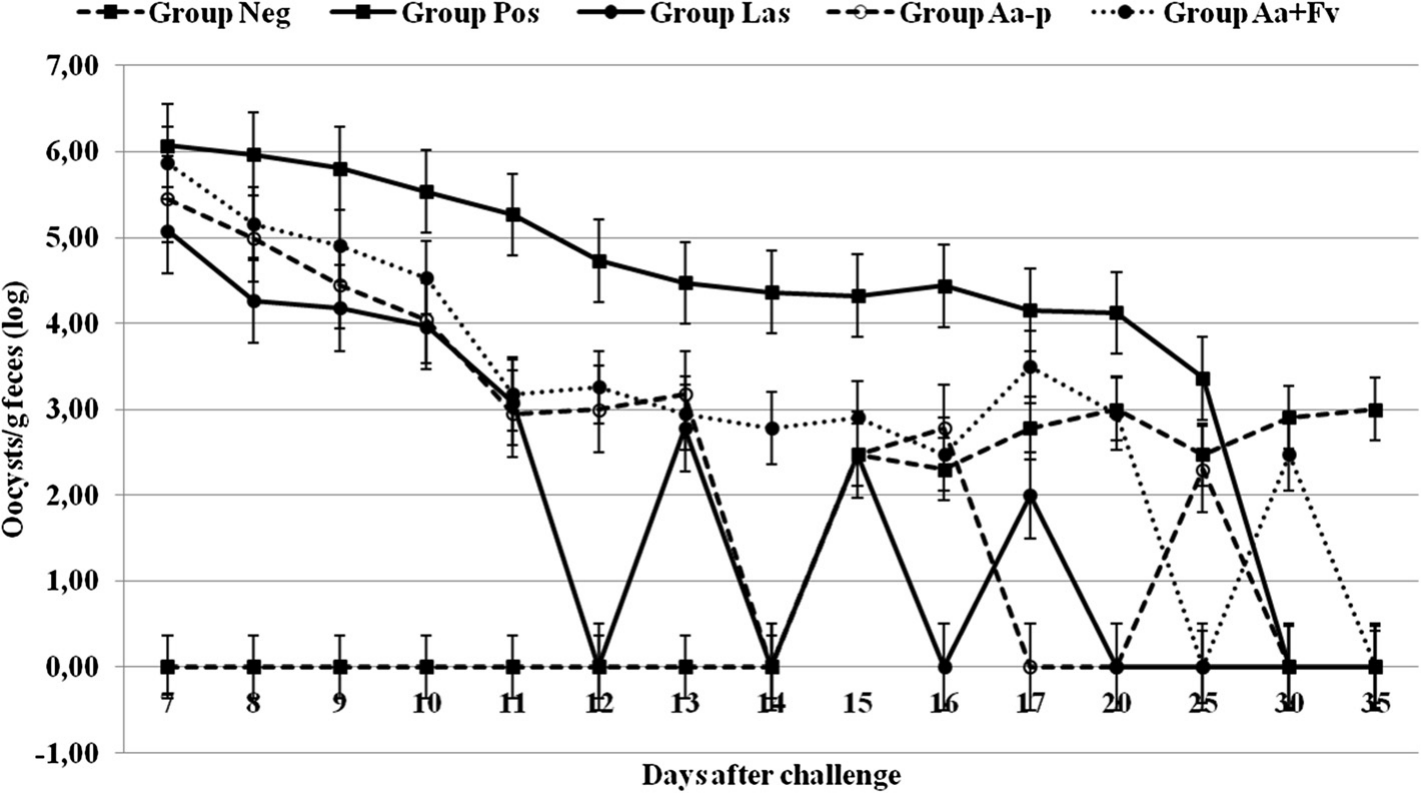
**Effect of *A. annua *** **(GERM07) (*Aa-p*) and mixed oil extracts of *F. vulgare *** **and *A. annua *** **(ROMN08) (*Fv + Aa-o*) on the fecal oocyst output, following experimental infection, of broiler chickens with high dose of *E. tenella *** **(1×10**^**4 **^**oocysts/chicken).**

A HPLC-UV chromatogram of the GERM07 *A. annua* powdered leaves can be seen in Figure 1. Triplicate analysis of the leaf material used in this study determined the percent (g/100 g dry weight) of sesquiterpenes produced in the leaves. Powedered leaves contained on average 0.75% artemisinin (Art), 0.18% dihydroartemisinic acid (DHAA), and 0.03% artemisinic acid (AA). Standard deviations for all sesquiterpene quantifications were 0.01%. Although deoxyartemisinin (DOArt) was also present in powdered leaves (Figure 2), DOArt lacks one oxygen molecule in the peroxide bridge found in artemisinin and is reported to have no biological activity [31].

### Clinical signs and mortality

Clinical signs and mortality are presented in Table 1. Briefly, first clinical signs appeared at 4 days post-challenge in both positive control and *Aa + Fv*-o groups, but signs ameliorated 9 days after challenge*.* 19/20 chickens from the positive control group developed clinical infection and 17/20 had bloody diarrhea. In group *Aa + Fv*-o 9/20 chickens were affected, and 5/20 had bloody diarrhea. Clinical signs appeared five days after challenge in 3/20 chickens in group *Las* and in 6/20 in group *Aa-p.* Bloody diarrhea was registered in 1/20 chicken in *Las* group and in 2/20 in *Aa-p* group. No clinical signs were registered in chickens of the negative control group. Mortality was registered only in the positive control group (7/20). Dead chickens from this group had a lesion score of 4.

**Table 1:** **Clinical signs (including weakness, inappetence, and polydipsia), (bloody) diarrhea and mortality in the experimental groups (20 chickens each) after *E. tenella *** **challenge (1×10**^**4 **^**oocysts)**

**Treatment group**	**Clinical signs ( *n */20)**	**Bloody diarrhea ( *n */20)**	**Mortality ( *n */20)**
*Neg*	0/20	0/20	0/20
*Pos*	19/20***	17/20***	7/20**
*Las*	3/20	1/20	0/20
*Aa-p*	6/20*	2/20	0/20
*Aa + Fv-o*	9/20**	5/20*	0/20

*P < 0.05; **P < 0.01; ***P < 0.001.

### Oocysts shedding, faeces and lesion scores

As shown in Figure 2, the chickens from groups *Aa + Fv*-o*, Aa-p, Las,* and *Neg* exhibited significantly reduced oocysts shedding when compared to the positive control group (*Pos*) (*P* ≤ 0.001). Only the group treated with essential oils of *A. annua* and *F. vulgare* shed significantly more oocysts (*P* ≤ 0.001) than the negative control group. Regarding the comparison of experimental groups with lasalocid group, no significant difference was recorded in oocyst counts. Nonetheless, the highest reduction in fecal oocysts output following *E. tenella* infection was for group *Las* (95.2%), followed by groups *Aa-p* (87.9%) and *Aa + Fv*-o (71.1%). The difference in the reduction of fecal oocysts output between *Las* and *Aa*-p groups was about 7.3% and between *Las* and *Pa + Fv*-o groups, 24.1%.

According to Table 2, the faeces scores in groups *Neg, Las, Aa-p* and *Aa + Fv*-o were lower than in positive control group, but significantly lower only in negative control group (*P* ≤ 0.01)*.*

**Table 2:** **Faeces score post infection (p.i.), lesion score, daily body weight gain (BWG) and feed conversion ratio (FCR) in chicken experimental groups given diets consisting of *Artemisia annua *** **leaf powder alone ( *Aa-p *) or combined with *Foeniculum vulgare *** **essential oil ( *Fv-o *), after infection with *Eimeria tenella *** **(1×10**^**4 **^**oocysts)**

**Treatment groups**	**Faecal score (average of days 4–9 p.i.)**	**Lesion score**	**BWG (g)**		**FCR**
			**Day 0-7**	**Day 0-35**	
***Neg***	0/0/0/0/0/0**	0/0/0/0/0/0/0/0/0/0***	41.4 ± 0.25***	69.6 ± 0.13**	1.86
***Pos***	2/4/4/2/3/0	4/3/3/4/3/4/4/3/4/0	33.5 ± 1.23	61.1 ± 0.92	1.98
***Las***	0/4/0/0/0/0*	0/0/0/3/1/1/1/1/0/0***	40.9 ± 0.33***	66.8 ± 0.17*	1.88
***Aa-p***	0/4/0/0/0/0*	1/0/0/2/2/2/0/4/3/0**	39.2 ± 0.41**	68.2 ± 0.20**	1.85
***Aa + Fv-o***	2/4/0/0/0/0	4/4/4/3/1/3/3/3/3/0	36 ± 0.99	64.1 ± 0.60	1.94

*P < 0.05; **P < 0.01; ***P < 0.001.

Negative group (*Neg*) was uninfected, untreated. Positive group (*Pos*) was infected, untreated. Birds that were infected and treated with lasalocid (*Las*) served as infected, treated controls.

The difference for lesion score was statistically significant (*P* ≤ 0.001) among experimental groups, with the highest lesion score in positive control group (3.2). However, only chickens treated with lasalocid (*P* ≤ 0.01), and *A. annua* (*P* ≤ 0.01) powdered leaves had a significantly lower lesion score than chickens in positive control group. Negative control group had no lesions. Also, there was no significant differences between groups *Las* and *Aa*-p (*P* = 0.28) (Table 2).

### Weight gain and feed conversion

The weight gain for chickens treated with the diet containing 1.5% of feed as *A. annua* leaf powder was higher than for chickens treated with 7.5% mixed essential oils of *A. annua* and *F. vulgare* in water, but lower for chickens in group *Las* (Table 2)*.*

Birds from the groups *Las* and *Aa*-p had weight gains that were 0.5 and 2.8 g/day lower than those of birds from the negative control group and 5.7 and 7.4 g/day significantly higher than those of birds in the positive control group (*P* ≤ 0.01), respectively.

Interestingly, birds in the negative control group had the highest weight gain (*P* ≤ 0.01), whereas birds from the group *Aa-p* had the best feed conversion, surpassing feed conversions observed for birds in the *Neg* group and also birds in the *Las* group (Table 2).

## Discussion

The purpose of our study was to evaluate the anticoccidial activity of *A. annua* and *F. vulgare* in chickens heavily infected (10,000 oocysts) with *E. tenella* using a floor-pen trial. Artemisinin is well known for its anti-malarial effect [32], but less is known about its activity against *Eimeria* spp. *A. annua* leaf powder was used at 1.5% (GERM07) in feed, or as an oil mixture, containing 1.875% of each *A. annua* (ROMN08) and *F. vulgare* essential oil in water, at 7.5%*.*

*A. annua* leaf powder protected 70% of infected chickens from mortality and pathological symptoms associated with *E. tenella* when added at 1.5% in daily feed. It significantly reduced fecal oocyst output (87.9%), lesion score (56.3%), while it increased weight gain (11.6%) and food conversion (6.6%) compared to infected and untreated chickens. Moreover, weight gain and feed conversion ratio were superior to the ones presented by the group treated with lasalocid. Although faeces scores between *A. annua* leaf powder and lasalocid were comparable, results for faecal oocyst output and lesion scores of the leaf powder group were not as good as the ones recorded for the lasalocid group. According to the interpretation of the anticoccidial-sensitivity tests, based on the reduction of the mean lesion score [33], *E. tenella* Houghton strain was fully susceptible (100%) to *A. annua* GERM07 as leaf powder.

Previously [34], when chicken were lightly infected with 1,500 oocyst of *E. tenella* (Houghton strain) and 1.5% *A. annua* leaf powder was added to the diet, the oocysts output was reduced by 90.8% and the lesion score by 55.5%, with similar reductions in oocysts output and lesion scores as obtained in the current study. The main difference between the current and the previous study was in the feed conversion ratio, which was 6.6% (current study), surpassing the 8.3% obtained in the previous study, and higher than positive control.

The few published studies on the effects of *A. annua* on coccidial infection varied greatly in results. On the plant side, this variation was probably a consequence of artemisinin concentration caused by either different chemotypes or seasonal variation [35], although different methods of drying can increase artemisinin concentration in leaves compared to freeze drying [36]. On the animal side, different results can be a consequence of routes of administration, experimental design (*Eimeria* species, oocysts dose, leaf powder ratio in feed, etc.), and different susceptibilities of *Eimeria* species to the treatment applied. In general, most of them provided a good control against *E. tenella* infection, but not for *E. acervulina* and *E. maxima*[18,37]*.*

The *A. annua* plant material designated as GERMAN07 used in this study contained 0.75% artemisinin (Art), 0.18% dihydroartemisinic acid (DHAA), and 0.03% artemisinic acid (AA). Although biological activity against several protozoan parasites has been shown for Art, the biological activity of DHAA and AA has not been proven or disproven against Eimeria spp., but AA had been reported to be effective against several bacteria and fungi in vitro [38]. The bactericidal activity of Art and AA at 500 μg/mL was approximately 50% of the activity of several commercial antibiotics tested at 1,000 μg/mL, except for *Staphylococcus aureus*[38]. However, it is unclear if the effect of the *A. annua* leaf powder used during this experiment can be attributed to Art only, or to a combination of the terpenes Art, DHAA, and AA, or yet, to a combined effect of all three with antioxidant flavonoids from the leaves, which are reported to potentiate the effects of artemisinin [32]. Flavonoids are well known for their antioxidant capacity due to their redox properties. Some flavonoids act on host-parasite interactions, and others disturb development or metabolism of protozoan parasites [39], including *Leishmania* spp. and *Trypanosoma* sp. [40].

It has also been demonstrated that artemisinin alters the process of oocyst wall formation resulting in an incomplete oocyst wall (organized at two opposite poles), with death of developing oocysts and reduction in the sporulation rate [41]. This alteration is caused by reduction of SERCA (sarco/endoplasmic reticulum calcium ATPase) expression in macrogametes, that plays a role in calcium homeostasis affecting the secretion of wall-forming bodies, a calcium-dependent mechanism [41].

Chickens treated with *A. annua* and *F. vulgare* as mixed oil extracts had 100% survival rates (as birds treated with lasalocid), reduced oocysts output (71.1% decrease), and improved faecal scores relative to untreated controls. The body weight gain and feed conversion ratio were higher than in positive control, but not statistically, and were lower compared with the other experimental groups, except the positive group. The difference in efficacy between powder and essential oil of *A. annua* could be attributed to the lack of artemisinin in the oil, and to the poor water solubility of essential oil components in commonly-used carriers like DMSO or Tween 80 [42]. Another possible explanation for the lower body weight gain of chickens treated with the oil mixture can be the possible toxic effects of phenolic compounds present in the essential oil mixture [43]. Components derived from the oil of *F. vulgare* seeds had acaricidal activity against *Tyrophagus putrescentiae*[44], antimicrobial and antioxidant capacity [24], but the antioxidant capacity of *A. annua* leaves is much higher than the antioxidant activity reported for oils because lipophilic leaf extracts (oils included) have only 5% or less of the total antioxidant capacity found in leaves [32]. Our study showed that chickens treated with the leaf powder had better lesion scores, weight gain, feed conversion ratio, less clinical signs and bloody diarrhea than chickens treated with the essential oil mixture, although both leaf powder and oil mixture protected chickens from mortality similarly and significantly better than found for positive control (infected/untreated) animals.

## Conclusions

Our results suggest that *A. annua* leaf powder (Aa-p), at 1.5% of the daily diet post-infection, can be a valuable alternative for synthetic coccidiostats, such as lasalocid. Infected chickens treated with Aa-p had 100% survival after infection with a high dose of *E. tenella*. In addition, chickens treated with Aa-p had significantly higher body weight gain compared to infected/untreated chickens. Further *in vivo* experimentation, also involving cost-benefit analysis, is needed to verify the feasibility of replacing synthetic coccidiostats with *A. annua* leaf powder for the control of coccidiosis in poultry production systems.

## Competing interests

The authors declare that they have no competing interests.

## Authors’ contributions

LD participated in the design of study, made the observations and analytical procedures, and drafted the manuscript. AG conceived the study, and participated in its design, helped to draft the manuscript, and performed the statistical analysis. JFSF performed the HPLC-UV analysis of *Artemisia annua*, helped to draft the manuscript, and revised the manuscript’s final version before resubmission. IAP and ID cultivated *A. annua* varieties, and obtained the powder and essential oil of *A. annua*. ID and MD helped with observations and analytical procedures. VM participated in the design of study, and helped to draft the manuscript. VC conceived the study, participated in its design and coordination, and revised the manuscript. All authors read and approved the final manuscript.
